# Atraumatic Fracture of Newer Generation Ceramic Head Three Days Post-op: A Case Report

**DOI:** 10.7759/cureus.75100

**Published:** 2024-12-04

**Authors:** Nicholas Brown, John Dundon

**Affiliations:** 1 Orthopedic Surgery, Orthopaedic Research Institute of New Jersey, Bridgewater, USA; 2 Orthopedic Surgery, Tri-County Orthopedics, Bridgewater, USA

**Keywords:** atraumatic fractures, ceramic-on-polyethylene, post-operative complication, revision hip arthroplasty, total hip arthroplasty (tha)

## Abstract

A 73-year-old female experienced an atraumatic fracture of a BIOLOX delta ceramic femoral head following uncomplicated right total hip arthroplasty using a ceramic-on-polyethylene bearing. The fracture occurred post-operatively, as revealed by radiography after the patient reported a clunking sensation and leg shortening. Revision surgery involved replacing the fractured head and liner with careful removal of ceramic debris. The patient recovered well with no further complications. This case highlights the rare occurrence of an atraumatic fracture in a newer-generation BIOLOX delta ceramic head. This suggests possible manufacturing defects or taper mismatches and emphasizes the importance of thorough implant evaluation.

## Introduction

Total hip arthroplasty (THA) is a highly common and successful procedure in the US with over 460,000 surgeries performed annually and expected to grow significantly by 2030 [1]. Complication rates remain low with the most common reasons for failure being dislocation, fracture, and infection [2]. With improvements in implant technology, failure rates from component failure have continued to improve over time. Improved polyethylene annealing and sterilization in inert gasses have led to improved wear characteristics and decreased component failure rates [3]. Bearing surfaces have continued to evolve over the years, decreasing the component failure rates.

The most common types of bearing surfaces applied in THA are metal-on-metal (MoM), metal-on-polyethylene (MoP), ceramic-on-ceramic (CoC), and ceramic-on-polyethylene (CoP). The most common bearing surfaces used in the US are MoP and CoP. MoP has shown higher wear rates in studies and is still susceptible to problems with trunnionosis, especially in large head sizes [4]. First-generation (referring to pre-1990) ceramic heads were made from alumina, and due to their brittle nature had issues with ceramic head fracture rates of 13.4% [5]. Zirconia and other metals have been added to ceramic components for their many benefits: increased hardness, scratch resistance, and lack of debris production [6]. One major drawback to the use of CoC bearings is the ceramic commonly breaks without plastic deformation due to its high elastic modulus [7]. Production processes have become more advanced, including the addition of zirconia to the BIOLOX delta heads, leading to increased durability and decreased fracture rates [8]. Methods and tools used to implant the prosthesis have also changed slightly over time, leading to increased survivability rates and decreased revision rates [9]. Despite the advancements in implant technology, ceramic head fractures still occur, although typically in CoC-bearing surfaces.

Herein we present a case of atraumatic ceramic head fracture from right THA in a CoP patient. To our knowledge, this is the first atraumatic case presented in the literature of a newer-generation ceramic head fracture with a polyethylene-bearing surface.

## Case presentation

**Figure 1:**
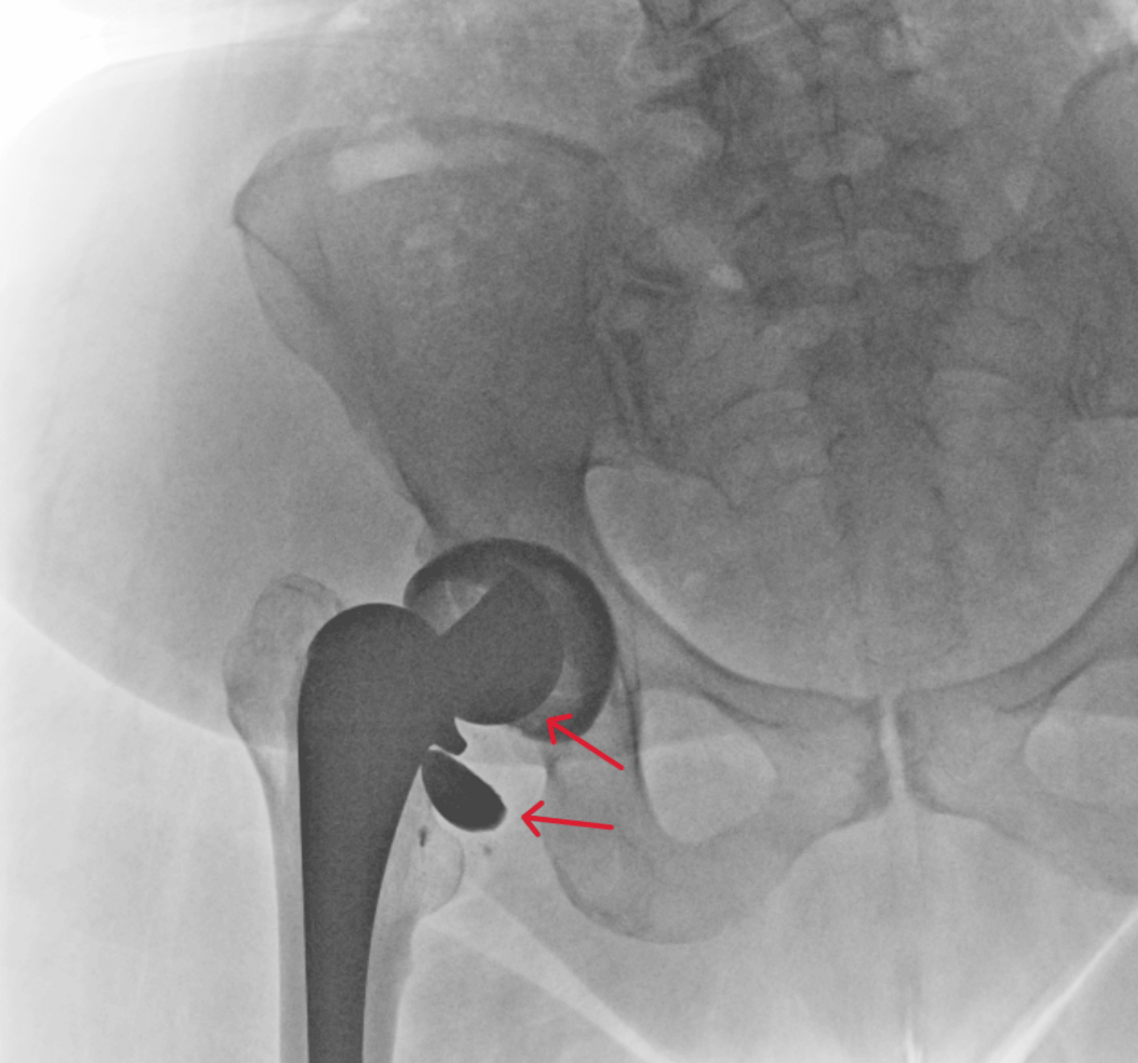
Anterior radiograph of the pelvis showing ceramic head fracture on the right side. Ceramic head fragments are identified by arrows.

**Figure 2:**
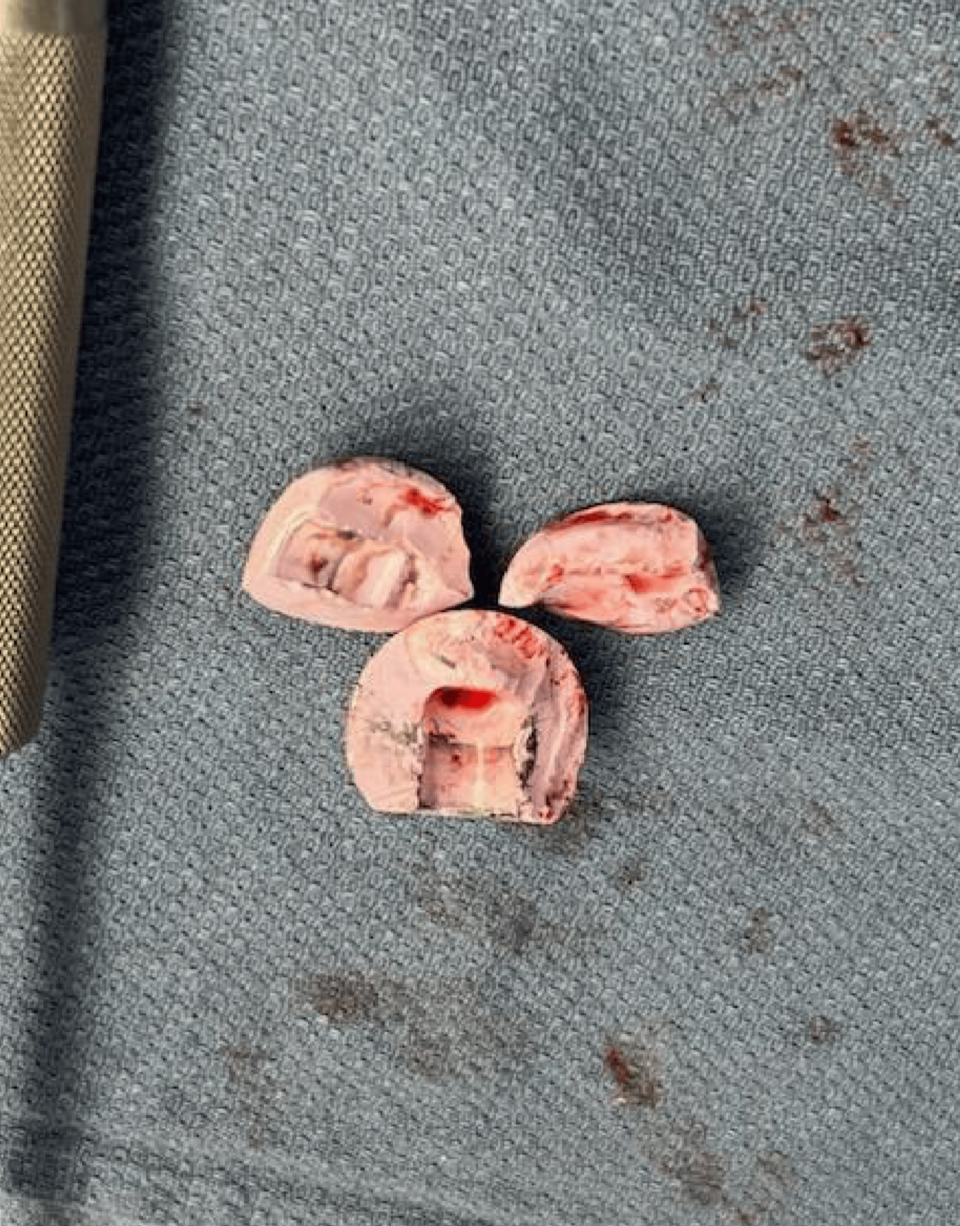
Ceramic head components following removal from the pelvis.

**Figure 3:**
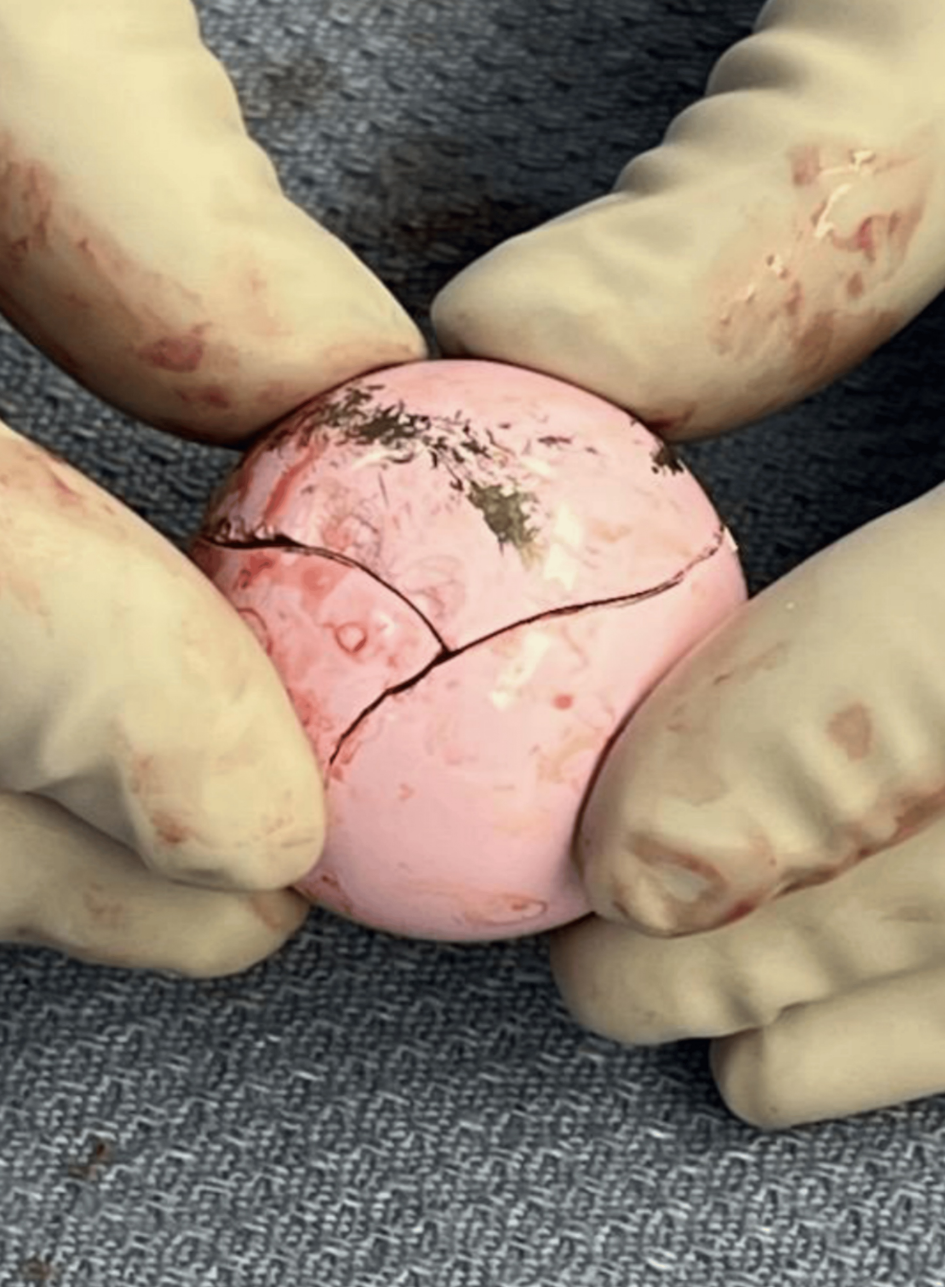
Proximal side of the fractured ceramic head components pieced together following removal.

The patient is a 73-year-old female who underwent an uncomplicated, anterior approach cemented right total hip arthroplasty. Intraoperatively there was no trauma and we had good exposure of the femur and trunnion. Before implantation of the femoral head, the trunnion was cleaned in a typical fashion. We implanted a 36+0 standard femoral head and the hip was stable through all ranges of motion. She was doing well and was ambulating with physical therapy without pain or complication. Early on while working with PT, she felt a clunk and noticed some shortening of the leg. Radiographs were taken and showed a fracture of the femoral head (Figure 1). She still had minimal pain, and there was no fall or trauma. She was subsequently taken back and revised with the femoral head and liner. The liner was destroyed by the trunnion buried into the polyethylene liner. We slowly removed all of the fragments from the ceramic head and pieced them back together to ensure all pieces were removed (Figures 2, 3). We also confirmed this with intraoperative fluoroscopy. Once this was done we removed the polyethylene liner and replaced this component with a new one. We replaced the head with a new 36+3 femoral head. During dissection to remove all of the ceramic debris further soft tissue release was required necessitating an increase in neck length for stability. The patient did well with physical therapy and was subsequently discharged to home. We followed her for three months postoperatively with no further complications and full range of motion without pain. The rest of her postoperative course was uneventful. The components were packaged and sent back to the manufacturer for analysis. The patient has a two-year follow-up with no pain and no complications. Informed consent was obtained from the patient for the publication of this case report and its images.

## Discussion

Ceramic head fracture following THA is a rare, but catastrophic complication. Rates have decreased following the switch from alumina to BIOLOX delta ceramic heads [7]. Our case represents the only case of an atraumatic fracture of a BIOLOX delta ceramic on a polyethylene hip in the literature. One major category of ceramic materials used in hip arthroplasty is alumina (Al_2_O_3_). Pure alumina heads are associated with a relatively high incidence of clinical fracture of 0.0201% when compared to the 0.0010% found with alumina matrix composite [10]. Although both material’s fracture rates are extremely low, the reliability is even better with alumina matrix heads. Squeaking is a commonly associated problem with hard-on-hard bearing hip replacements and can be caused by improper fluid film lubrication in the bearing. Causes of this include stripe wear, edge loading, introduction of unwanted particles, and ceramic fracture [11]. The rubbing of non-lubricated parts may produce sounds audible to humans, however, this complication has not been directly associated with pain. 

Stripe wear has also been found to play a role in the durability of ceramic bearings. Stripe wear is caused by a microseparation of the femoral head leading to impingement during different parts of the gait cycle. It has also been found that stripe wear may occur due to a lack of lubrication during edge loading [11]. As stripe wear begins to form, it becomes more likely for the problem to progress faster with time. The wettability of the surface has been found to have an impact on the durability of prosthetic materials. Materials with high wettability allow a fluid to remain in contact with a surface. Ceramic materials are often harder and smoother than their metal alloy counterparts, allowing for better lubrication [12]. High wettability is associated with the promotion of material-tissue interaction, but may also be associated with increased corrosion, therefore a material with well-balanced wettability characteristics must be used [13]. 

Although ceramic’s hardness is what makes it so good for use as a material in prosthetics, it is also one of its largest downfalls due to the likelihood of fracture with little plastic deformation due to its high elastic modulus [8]. Increased material hardness is desired when making a prosthetic, especially for younger patients, due to its linkage to better corrosion resistance and overall toughness [14]. Due to ceramic’s naturally high modulus of elasticity, it is naturally poor at dampening impacts made to the material and can lead to lubrication and durability issues in the future.

BIOLOX delta ceramic heads were first introduced in 2003 and the delta-delta hip bearing was introduced in 2010. The ceramics used were combined with zirconia and several other materials to improve material hardness and further decrease the possibility of crack formation [15]. This new ceramic was also produced as an attempt to minimize the production of minute commutia following a fracture by increasing bend strength [16]. Recent registry data reveals a fracture risk for BIOLOX delta of 0.1% [17]. This rate may be even lower for ceramic-on-polyethylene bearings, considering the data used was solely based on ceramic-on-ceramic bearings [18]. With the low fracture rate of fourth-generation ceramic-on-ceramic bearings, other components are being analyzed for their contribution to revision rates. Ceramic surfaces also minimize or even negate the risk of trunnionosis, which has been found to account for up to 3% of all revisions [19]. Wear patterns associated with ceramic-on-ceramic bearings have even been found to have improved performance during stop-dwell-start motions when compared to ceramic-on-polyethylene and metal-on-polyethylene bearings [20]. 

The literature contains many examples of traumatic ceramic head fractures in THA but does not contain many non-traumatic examples. This case is even more rare since the fracture occurred days following the procedure. In discussion with the company post-implant analysis, it was determined that this was likely a machining defect. There was either a slight defect in the ceramic head or a slight mismatch in the Morse taper leading to catastrophic failure. Another option would be a slight defect in the femoral stem taper but we had no further issues with the new ceramic head so that was excluded. It is also possible that the head was placed malaligned during impaction but there were no complications during surgery and we had a good fit intraoperatively. No other common causes of non-traumatic head fracture seem to be validated due to the paucity of these cases in the literature.

## Conclusions

This case study highlights a rare instance of atraumatic ceramic head fracture in a CoP-bearing surface following THA. Despite advancements in ceramic technology, including the development of BIOLOX delta heads that are engineered for increased fracture resistance, this case demonstrates that ceramic fractures remain a possible complication. Although the fracture may have resulted from a machining defect or mismatch at the trunnion interface, the underlying cause remains speculative due to the low incidence of atraumatic ceramic fractures in THA. This case underscores the importance of ongoing advancements in implant manufacturing processes and material quality control to further reduce these rare complications. Overall, while THA continues to demonstrate excellent outcomes with low complication rates, this case emphasizes that vigilance and refinement in implant technology remain critical for optimizing patient outcomes.
